# Self-supervised representation learning for surgical activity recognition

**DOI:** 10.1007/s11548-021-02493-z

**Published:** 2021-09-20

**Authors:** Daniel Paysan, Luis Haug, Michael Bajka, Markus Oelhafen, Joachim M. Buhmann

**Affiliations:** 1 Department of Computer Science, ETH Zurich, Zurich Switzerland; 2 Division of Gynecology Department OB/GYN, University Hospital, Zurich, Switzerland; 3 VirtaMed AG, Schlieren, Switzerland

**Keywords:** Self-supervised Learning, Representation Learning, Unsupervised Learning, Surgical Activity Recognition, Deep Learning, Probabilistic modeling

## Abstract

*Purpose:* Virtual reality-based simulators have the potential to become an essential part of surgical education. To make full use of this potential, they must be able to automatically recognize activities performed by users and assess those. Since annotations of trajectories by human experts are expensive, there is a need for methods that can learn to recognize surgical activities in a data-efficient way. *Methods:* We use self-supervised training of deep encoder–decoder architectures to learn representations of surgical trajectories from video data. These representations allow for semi-automatic extraction of features that capture information about semantically important events in the trajectories. Such features are processed as inputs of an unsupervised surgical activity recognition pipeline. *Results:* Our experiments document that the performance of hidden semi-Markov models used for recognizing activities in a simulated myomectomy scenario benefits from using features extracted from representations learned while training a deep encoder–decoder network on the task of predicting the remaining surgery progress. *Conclusion:* Our work is an important first step in the direction of making efficient use of features obtained from deep representation learning for surgical activity recognition in settings where only a small fraction of the existing data is annotated by human domain experts and where those annotations are potentially incomplete.

**Supplementary Information:**

The online version contains supplementary material available at 10.1007/s11548-021-02493-z.

## Introduction

The increasing importance of computer-assisted surgery has not only fundamentally changed the competencies surgeons require, but also fueled the development of novel educational methods [4]. For example, virtual reality-based simulators enable surgeons to practice surgical procedures and skills in safe, diverse and highly realistic environments [3]. In order to tap the full potential of such simulators as educational instruments, they must be able to provide automatic performance assessment and feedback. An important step towards this goal is the automatic recognition of the surgical activities performed by users.

Most recent progress towards surgical activity recognition relies on the use of deep learning [1, 8, 11]. However, these methods have the drawback that their training requires extensive amounts of annotated data. While virtual reality-based simulators record surgical motion data automatically, the activity annotations must be provided *manually* by domain experts. As a consequence, obtaining sufficient amounts of annotated training data for complex surgical procedures involves a time-consuming labeling process which is often prohibitive in practice. This explains the need for surgical activity recognition methods that work when annotated data are scarce.

So far, little work has been conducted in this direction. Existing works can be divided into two distinct categories depending on the data modality they focus on: a) video-based [5, 12, 22] and b) motion-based surgical activity recognition [9, 10]. To the best of our knowledge, studies combining sensor-based motion and video data have not been conducted in the context of surgical activity recognition with scarce annotations. The present paper addresses this research gap. We propose a method to learn representations from video data and to use those in combination with sensor-based motion data to improve surgical activity recognition without the need for expensively annotated data.

We make the following contributions:

1. We present a method for semi-automatically extracting features from surgical video data containing information about relevant events in surgical trajectories. Specifically, we describe a deep learning architecture that learns spatio-temporal representations from surgical videos in a self-supervised way, and we propose a procedure for the semi-automatic analysis of the learned representations.
2. We show that features extracted via this method can be used to significantly improve the performance of unsupervised surgical action recognition approaches requiring structured (categorical or continuous) input. To this end, we use such features as observables in a hidden semi-Markov model for surgical activity recognition. We provide empirical evidence for the potential of this approach by testing it on a novel data set of trajectories collected on a hysteroscopy simulator.

## Methods

Our objective is to learn a model that predicts activity labels for trajectories collected on a surgical training simulator. Every trajectory corresponds to an individual simulator run in which a surgeon performs a fixed surgical task. While the specific task for which we developed our model is a myomectomy task on a hysteroscopy simulator, our methodology can also be applied to the analysis of other scenarios.

We model trajectories as pairs $(\varvec{X}, \varvec{Z})$, where $\varvec{X} = \{\varvec{x}_t\}_{t=1}^{\tau }$ is a sequence of video frames and $\varvec{Z}=\{\varvec{z}_t\}_{t=1}^{\tau }$ is a sequence of sensor data describing, e.g., motions or tool usage. Our goal is to classify each step *t* as belonging to a subtask (or *activity*) of the surgical procedure from a finite set of possible activities

$$\begin{aligned} \mathcal {S} = \{activity_1, ~\dots , ~ activity_n\} \end{aligned}$$

defined by a hierarchical task decomposition model (HTDM) devised by a domain expert for the specific task. That is, we want to predict for a trajectory represented by $(\varvec{X}, \varvec{Z})$ a sequence $\varvec{A}=\{a_t\}_{t=1}^{\tau }$, where $a_t \in \mathcal {S}$ is the activity at time *t*.

Our approach first uses the video data $\varvec{X}$ to construct sequences of spatio-temporal representations $\varvec{R} = \{\varvec{r}_t\}_{t = 1}^{\tau }$ via self-supervised training of a deep encoder–decoder model on a pretext task that is closely related to the activity recognition task. In a second step, we extract from those representations sequences of features $\varvec{F}$ which carry useful information related to the activity recognition task. Finally, we train a hidden semi-Markov model whose observables are constructed from $\varvec{Z}$ and $\varvec{F}$ to predict the sequence of activities $\varvec{A}$ for the trajectory.

### Self-supervised representation learning

Self-supervised visual representation learning has recently evolved to an active area of machine learning research [14, 16, 17, 23]. The idea of such approaches is to train a deep neural network on a *pretext task*, i.e., an auxiliary supervised learning problem for which the required labels can be extracted automatically from unlabeled data, thus avoiding the need for expensive manual labeling. The representations learned in this manner are then used for the downstream task that one ultimately tries to solve.

**Remaining surgery progress pretext task**

Inspired by [20] and [21], the pretext task we use is the estimation of the remaining surgery progress (RSP)

1 $$\begin{aligned} y_t = 1-\frac{t}{\tau }, \end{aligned}$$

**Fig. 1:**
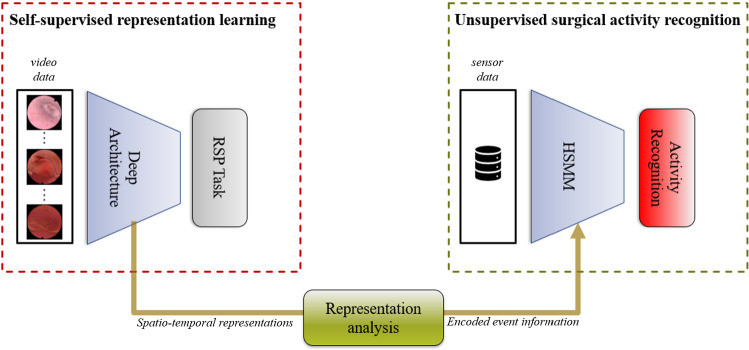
Summary of the proposed unsupervised activity recognition approach using self-supervised representation learning to allow for the modeling of different data modalities, i.e., the use of video and sensor data

i.e., the remaining surgery duration relative to the total time $\tau$ of the surgery. The choice is based on our hypothesis that in order to estimate the overall progress of a surgical procedure a deep architecture applied to that task must implicitly recognize important milestone events like, e.g., the end of the initial diagnostic activities, which is valuable information for activity recognition.

**Encoder–decoder architecture** To predict the RSP (1) from video data, we apply a deep encoder–decoder architecture. Such models have been shown to be able to learn abstract spatio-temporal representations of the input data [21]. The architecture consists of an *encoder*, which is a CNN that computes spatial representations $\{\varvec{\tilde{x}_t}\}_{t=1}^{\tau }$ based on the visual information $\{\varvec{X}_t\}_{t=1}^{\tau }$. These representations are then processed by a *decoder*, which is a recurrent neural network that computes a condensed representation of the history of the trajectory that is being processed. Finally, the output of the decoder is mapped by fully connected layers to the predicted RSP values $\{y_t\}_{t=1}^\tau$.

### Representation analysis

The structure of the encoder and decoder described above enables the deep model to learn representations of the surgical video data at various abstraction levels. Our goal is to extract information from these representations that can be used to recognize events relevant for activity recognition, such as the beginning or end of surgery phases or activities. To that end, we consider the outputs of individual layers of the encoder and decoder, as well as the activations of the output gates of the decoder, as suggested by [19]. This yields a multivariate time series $\{\varvec{r}_t\}_{t=1}^{\tau }$ for each of the surgical trajectories. We then proceed as follows:

1. We apply a change point detection algorithm to each $\{\varvec{r}_t\}_{t=1}^{\tau }$ to obtain a collection of change points $\{t_1, \dots , t_n\}$.
2. We perform a visual analysis of the video data to detect consistent links between the detected change points and relevant events.
3. We analyze the representations $\{\varvec{r}_{t_i} \}_{i=0}^{n}$ at the detected change points to identify components that are particularly strongly associated with the relevant events.
4. We extract features from those components that are then later on used for the activity recognition.

**Fig. 2:**
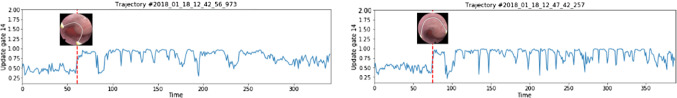
Plot of the activation of the update gate 14 for which the first increase encodes the end of the diagnosis here shown for two randomly chosen trajectories

### Surgical activity recognition with HSMMs

The features extracted from the video and sensor data $(\varvec{x}_t, \varvec{z}_t)$ are used as input for a hidden semi-Markov model that is used to predict the most probable sequence of activities $\varvec{A}$. A *hidden semi-Markov model* (HSMM) is a statistical model describing a system with a time-dependent “internal” state $s_t$. The state cannot be observed directly, but it can only be inferred through noisy observations $o_t$ that the system emits. Given the noisy observations, the model can be used to infer the sequence of states (or activities) that best explain the observations. In the Supplemental Information, we provide a more formal definition of a HSMM.

A summary of our proposed unsupervised activity recognition approach using self-supervised representation learning is given in Figure 1. Details on how we implement the approach in our concrete application setting are given in Section 4-6.

## Data

### The VRSHM dataset

To gather the data with which we tested our approach for activity recognition, we conducted a study in which ten surgeons produced three to four trials of a hysteroscopic myomectomy task on a virtual reality-based simulator. In total, 38 trials were conducted and trajectories were recorded by the simulator for each of the trials. We refer to the collection of these trajectories as the *virtual reality simulated hysteroscopic myomectomy* (VRSHM) data set.

Each trajectory consisted of sensor and simulated video data. A measurement of the sensor data was recorded whenever one of the features changed. The video sequences were recorded at 40 frames per second, with each frame having a resolution of $512\times 512$ pixels. The average video duration is about $248 \ (\pm 110)$ seconds. The VRSHM data set captures the variation in surgical procedures due to heterogeneous patient and clinician populations well and thus allows the validation of theoretical approaches in a setting, which realistically reflects the challenges of surgical activity recognition in complex domains.

### The hierarchical task decomposition model

A hierarchical task decomposition model (HTDM) was constructed by a domain expert. This model defines the set of possible activities which can be performed during a hysteroscopic myomectomy and the possible transitions between these activities. We used a simplified version of the model that divides a hysteroscopic myomectomy into a diagnostic part in which the uterus is inspected and an operative part in which a myoma is removed. It then further subdivides the operative part into steps as illustrated in Supplemental Figure S1. Jointly, the model defines the set of activities that we considered in this work and which are further described alongside the overall procedure in Supplemental Table S1.

One of the sequences in the VRSHM dataset was annotated with respective activity labels by the domain expert. These annotations were only used for the unsupervised activity recognition approach during the hyperparameter initialization and model validation as further described in Section 6. Additionally, manual annotations were obtained for two key activities (*diagnosis* and *handle chips*) from the domain expert for 18 trajectories in total. Those annotations were only used for the performance assessment of the trained models.

## Self-supervised representation learning

We now describe how we selected and trained a model to predict the remaining surgery progress (RSP) for the myomectomy trajectories in the VRSHM dataset.

### The encoder–decoder model

Motivated by computational limitations and the size of our dataset, we chose relatively shallow architectures for the encoder part of our RSP prediction model. Specifically, we used Alexnet and Resnet18 instances pretrained on Imagenet [7]. We replaced their output layer with a fully connected layer that a) consisted of the number of neurons expected as input for the serial decoder model or b) a single output neuron for the CNN baseline models. For the encoder–decoder architectures, we appended a three-layered MLP where the final layer of each model consisted of single output neuron. The first two layers consisted of the same number of neurons as the final layer of the serial decoder (GRU or LSTM) to which a Parametric Rectified Linear Unit (PReLU) activation was applied.

To get outputs $\hat{y} \in [0,1]$, the interval in which the true RSP labels take values, we applied a sigmoid activation to the output of the final layer. The architectures were trained to minimize the mean absolute error (MAE) using the Adam [15] and RMSProp [6] optimizers with the default parameters of the Pytorch implementation [18] and a learning rate of $10^{-5}$.

We trained the models on whole surgical video sequences that we downsampled to a resolution of $256\times 256$ pixels at a frequency of 2 fps. We performed ten-fold cross-validation to identify the optimal hyperparameter configuration of the models. The details of the procedure including a list of the analyzed configurations are given in the Supplemental Information. Supplemental Table S3 summarizes the model configurations of the best performing models that we identified during the hyperparameter search.

## Analysis of learned representations

The high performance of our RSP prediction model suggested that it had learned spatio-temporal representations that contained information about the progress of the surgery. We now describe how we extracted from these representations information useful for recognizing events in the surgical task.

We performed the semi-automatic study described in Section 2.2. Concretely, we used the PELT algorithm [13] to detect change points formally described in the Supplemental Information. For the measure of fit, we chose the kernelized mean change [2]. We set the penalty parameter such that on average 10 robust change points were detected to limit the complexity of the manual analyses of those.

Two events that were consistently identified were a) the end of the initial diagnostic activity and b) the end of the first cut. The former is typically marked by the first extension of the hysteroscope and thus its first appearance in the camera picture. The latter is well visible by the loop no longer having contact with the tissue after cutting through it for the first time.

Our analysis also showed that update gate 14 of the first GRU layer of the serial decoder showed a significant increase in its activation as soon as the video suggested the diagnosis had ended. Based on this information, we constructed an observable indicating if the diagnosis has ended, see Figure 2.

## Activity recognition

The analysis in Section 5 suggests that our deep encoder–decoder architecture learned representations that could be used to recognize the end points of the *diagnosis* and the initial *cutting* activities. In particular, the former is not well captured by the sensor data. Thus, we hypothesized that the use of features encoding such information in addition to the sensor data could improve the unsupervised activity recognition approach using HSMMs. To verify this empirically, we compared the performance of HSMMs whose observables were defined using only sensor data with HSMMs whose observables also included such features learned in the self-supervised setting on the task of activity recognition on the VRSHM data set.

In Section 6.1, we will describe the components that all HSMMs we tested had in common and then provide a detailed description of the best-performing HSMMs in Section 6.2.

### HSMM components

**States, transition and duration models.** The state space for all HSMMs was the set of activities describing a hysteroscopic myomectomy as defined by the HTDM shown in Supplemental Figure S1. These activities were the *diagnosis*, *position hysteroscope*, *cutting*, *coagulation*, *clear view* and *handle chips*. Accordingly, we defined the transition model and the initial state distribution by categorical distributions. For the duration model, we used negative binomial distributions to account for the large intersample variance of the VRSHM data set with respect to the duration of the different activities.

**Observables and emission models.** We constructed observables from the sensor data, e.g., describing the state of certain pedals, valves or position data. A complete list is given in Supplemental Table S3. We found that models that used only categorical input features, some of which were derived from continuous variables such as the position of the hysteroscope, performed better than models that used only continuous or both continuous and categorical input features. Thus, we limited the scope of our consecutive analysis to HSMMs with discrete and finite observation spaces. We defined the emission models based on the used set of observables by a collection of activity-dependent categorical distributions. In our experiments, we only varied the set of used observables and hence, the emission model, and we kept the remaining model components constant. In this way, we could ensure that the differences of the results of the experiments could be fully explained by the different choices of observables and were not confounded by other design choices (such as those related to the duration or transition models).

**Initialization of model components.** We initialized the transition models, the duration models and the initial state distributions manually with the assistance of the domain expert. For the emission distributions, we used an automated initialization approach: We derived the maximum-a-posteriori estimates for the components of the emission model using the single fully annotated sequence and a conjugate Dirichlet prior that served as a regularizer.

### Comparison of best-performing HSMMs

To compare the performance of the different HSMMs, we used these models to infer the most probable activity sequences for the 18 surgical trajectories of medium length. We limited the study to those sequences a) to ensure that the duration distributions could be approximated sufficiently well by unimodal distributions and b) because only for those trajectories manual annotations by the domain expert were available. We then compared the results qualitatively, by inspecting how plausible the resulting segmentations were in view of the video data. We also provide a partial quantitative evaluation by computing mean intersection-over-union (IoU) scores for two key activities for which we had ground truth labels, namely the initial *diagnosis* and the final *handle chips* activities, see Table 1.

In particular, we compared the SensorHSMM, the best-performing model using only observables constructed from sensor data, with the UpdateGateHSMM, a model that used features derived from self-supervised representation learning.

**Table 1:** The mean IoU (mIoU) scores (higher scores are better) evaluated on the 18 trajectories for which ground truth annotations for two key activities were obtained from the domain expert

**Model**	**mIoU**:*diagnosis*	**mIoU**: *handle chips*
SensorHSMM	0.7409 (0.2809)	0.1183 (0.2270)
UpdateGateHSMM	**0.8466 (0.1846)**	**0.7369 (0.2109)**

**Fig. 3:**
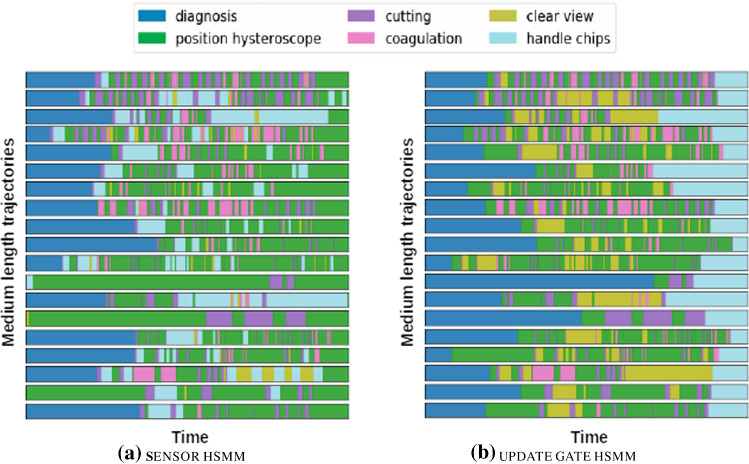
Visualization of the segmentation of the 18 trajectories of medium length using the SensorHSMM and the UpdateGateHSMM. Only the latter makes use of features derived from self-supervised representation learning

**Fig. 4:**
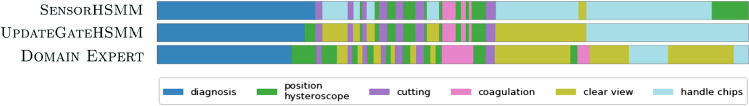
Comparison of the segmentations for the annotated trajectory of the surgery

SensorHSMM **.**

The segmentation of the sequences produced by the fitted SensorHSMM provided a reasonable explanation of the observation sequence with respect to the recognized activities. The *cutting* and *coagulation* activities were well identified by the model. However, it was not able to distinguish well between the *handle chips* and *position hysteroscope* activities. Additionally, for some trajectories the model misclassified the starting activity, which in all cases was a *diagnosis* as annotated by the domain expert. Figure 3 shows the segmentations produced by the SensorHSMM.

UpdateGateHSMM **.** The use of an indicator if the diagnosis activity has ended derived from the learned spatio-temporal representations (see the description in Section 5) led to a superior segmentation of the individual activities. We refer to the corresponding HSMM as the UpdateGateHSMM. Figure 3 shows the segmentation of the trajectories produced by the fitted UpdateGateHSMM. Compared to the SensorHSMM, it was more sensitive to the differences between the *handle chips* and *position hysteroscope* activities. Moreover, the model correctly recognized that all of the trajectories started with an initial *diagnosis* activity.

The superiority of the UpdateGateHSMM also becomes apparent when assessing the mean IoU of the segmentation with respect to the two labeled activities as shown in Table 1. Both the *handle chips* and *diagnosis* activities were better recognized by the UpdateGateHSMM compared to the SensorHSMM.

Comparing the segmentation by the UpdateGateHSMM to the manual annotations of the domain expert for the single completely annotated sequence further emphasizes the value of incorporating the information derived from our self-supervised learning approach. Figure 4 shows that the segmentation of the UpdateGateHSMM better matches the manual annotations than the one by the SensorHSMM.

The improved sensitivity of the UpdateGateHSMM with respect to the *diagnosis* activity compared to the SensorHSMM can be explained by the fact that the spatio-temporal representations capture a key indicator for the end of the diagnosis: the first extraction of the hysteroscope and thus its first appearance in the video. This indicator is also used by the domain expert during the manual segmentation and improves the separability of the *diagnosis* and *position hysteroscope* activities, for which the tool usage and hence, the sensor measurements are often very similar (as is the case for the *clear view*, *coagulation* and *handle chips* activities). The latter also explains, e.g., why both HSMM models fail to identify the final coagulation activity.

## Discussion

We introduced an approach for using spatio-temporal representations learned by training a deep encoder–decoder model on a pretext task as observables in a HSMM used for unsupervised surgical activity recognition. The experiments with our VRSHM data set show that the self-supervised representation learning could substantially improve the results of activity recognition. The high intersample variance, the small sample size and the lack of sufficient activity annotations of the used data set do not allow for more general statements. Despite these limitations, we believe that the results are an indication for the potential of self-supervised representation learning to enhance unsupervised activity recognition approaches in complex domains like medicine and healthcare in general.

To provide further evidence of the potential of such approaches, we believe that future work should focus on the use of larger data sets with more annotated sequences. This would enable a quantitative assessment of the benefit of self-supervised representation learning for unsupervised activity recognition. As mentioned before, obtaining such surgical data is often expensive. Thus, it is important that future work puts a stronger focus on unsupervised activity recognition approaches and rely on annotated data mainly for model validation purposes in complex domains. Our work provides the first study combining different data modalities in such a context and indicates the large potential of such multi-modal approaches. We hope that the research community will join us studying methods that reduce the dependency on manually labeled data in the context of the important task of surgical activity recognition.

## Supplementary Information

Below is the link to the electronic supplementary material.

Supplementary material 1 (pdf 345 KB)

## Declarations

### Availability of data and material

The data to reproduce the experiments can be made available upon request.

### Code availability

The code required to reproduce the experiments can be made available by the authors upon request.
