# Supplementary material for: Self-supervised representation learning for surgical activity recognition
Source: Int J Comput Assist Radiol Surg. 2021 Sep 20;16(11):2037–44. doi: 10.1007/s11548-021-02493-z (PMC8589823; doi:10.1007/s11548-021-02493-z)
Supplement: Supplementary file 1 — Supplementary material 1 (pdf 345 KB) [file 11548_2021_2493_MOESM1_ESM.pdf]

Supplemental Information

Self-supervised Representation Learning for Surgical Activity Recognition

Daniel Paysan *[1], Luis Haug *[1], Michael Bajka[2], Markus Oelhafen[3], and Joachim M. Buhmann[1]

[1]Dep. Computer Science, ETH Zurich, Zurich, Switzerland
[2]Division of Gynecology, Dept. OB/GYN, University Hospital, Zurich, Switzerland
[3]VirtaMed AG, Schlieren, Switzerland

Description of the surgical procedure

Figure S1 shows the simplified version of the hierarchical task decomposition model that we refer to in Section 3.2 of the main paper. It describes the basic procedure of a hysteroscopic myomectomy in a flow chart. A description of the individual activities is given in Table S1.

**Activity**	**Description**
diagnosis	The summary of multiple diagnostic activities, which includes the inspection of the entire uterine cavity and the inspection of the myoma.
position hysteroscope	The activity of positioning the hysteroscope, which is done to prepare for a *cutting*, *coagulation* or *handle chips* activity.
cutting	The activity of cutting off part of the tissue.
coagulation	The activity of staunching a bleeding.
clear view	The activity of flushing the system to remove blood that opacifies the view.
handle chips	The activity of collecting and extracting the pieces of tissue ("chips") that have been cut off.

Tab. S1. Description of the activities performed in a hysteroscopic myomectomy

*contributed equally to this work

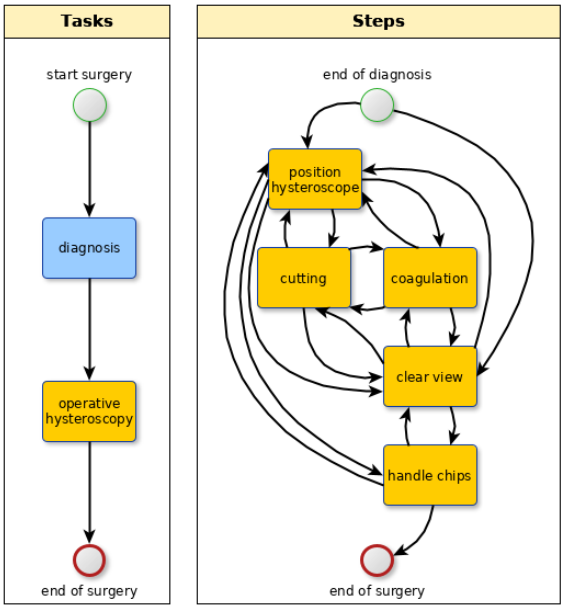

Fig. S1. Simplified hierarchical task decomposition model (HTDM) of a hysteroscopic myomectomy that defines the activities performed during the procedure. The *diagnosis* task jointly with the steps of the operative part of the procedure form the set of activities that we considered in the context of this work.

Hyperparameter optimization for the self-supervised representation learning.

Here we provide additional details regarding the hyperparameter optimization for the spatio-temporal models described in Section 4 of the main paper. We performed ten-fold stratified cross-validation approach for the hyperparameter optimization. To that end, we divided the surgical trajectories into three categories: Similar to [5], the trajectories whose duration was less than the lower quartile value of the empirical duration distribution of all trajectories in the data set were classified as *short* trajectories; those lasting longer than the upper quartile value were classified as *long* trajectories, and the remaining ones as *medium* trajectories. The data set was split such that the relative abundance of sequences of these three classes were approximately the same in each fold. Thereby, we ensured that the different folds were comparable despite the large intersample variance in the data set. The length of a sequence served as a simple proxy for the comparability of different surgical trajectories.

Finally, at each iteration the training portion of the data (i.e., the data without the fold held out for cross-validation) was additionally split into the set of sequences to train the model (80%) and a validation set (20%). The validation set was used to monitor the performance of the model within each iteration of the cross-validation and to e.g. early stop the training if the validation score has not improved for 20 epochs. We trained every model until convergence of the validation loss, which took up to 300 epochs.

The hyperparameter settings that we analyzed to identify the deep architecture that performed best on the VRSHM data set are summarized in Table S2. The performances of the five best encoder-decoder models among those we analyzed, as well as the performances of two baseline models; a Alexnet[4] and a Resnet18 [2] trained on single frames from the video sequences, are shown in Table S3.

**Hyperparameter**	**Description**	**Searched Domain**
`encoder_model`	The chosen pretrained CNN model architecture	{Alexnet, Resnet18}
`weight_decay`	The amount of l_2 weight regularization applied during training	10^k for $k \in \{-5, ..., 0\}$
`cnn_dropout`	The amount of dropout applied to the output of the CNN architecture	d^{-1} for $d \in \{0, ..., 5\}$
`cnn_features_dim`	The number of output dimensions of the encoder model	2^k for $k \in \{6, ..., 12\}$
`decoder_model`	The chosen type of the serial model architecture for the decoder	{LSTM, GRU}
`hidden_size`	The number of LSTM/GRU cells in each layer of the LSTM/GRU decoder	2^l for $l \in \{4, ..., 8\}$
`decoder_num_layers`	The number of layers of the LSTM/GRU decoder	$\{1, .., 4\}$
`decoder_dropout`	The amount of dropout applied between the layers of the LSTM or GRU in the decoder	d^{-1} for $d \in \{0, ..., 5\}$

Tab. S2. Overview of the hyperparameters that were tuned during the hyperparameter search. The hyperparameters in the bottom part of the table are not defined for the baseline models, but only for the encoder-decoder architectures.

Model	GRU/LSTM	Optimizer	CV MAE	CV STD
Alexnet	-	Adam	0.13360	0.01959
Resnet18	-	Adam	0.13440	0.01984
AlexnetGRU	512‖128‖128‖128‖128	RMSProp	**0.09929**	0.02584
AlexnetGRU	256‖32‖32	RMSProp	0.10529	0.02517
AlexnetLSTM	512‖128‖128‖128‖28	RMSProp	0.11845	0.04485
AlexnetLSTM	128‖128	RMSProp	0.11466	0.04615
AlexnetAttGRU*	512‖128‖128‖128‖128	RMSProp	0.11974	0.03332

Tab. S3. Performance overview of five of the best performing model configurations analyzed during the hyperparameter search using the discussed ten fold stratified cross-validation approach. All shown encoder-decoder models were trained using a l_2 weight regularization of 10^{-5}. The first two models are the CNN models that served as baselines. A '*' indicates the application of an attention layer to the output of the CNN encoder as part of the architecture. The GRU/LSTM column provides information regarding the architecture of the respective GRU/LSTM layers of the encoder-decoder models by describing the number of units in the different layers.

Pruned Exact Linear Time (PELT) algorithm

To automatically identify important change points in the multivariate time series given by the learned spatio-temporal representations as described in Section 5 of the main paper we applied the PELT algorithm. We here provide a formal description of the respective algorithm.

Algorithm 1: PELT algorithm by [3]

input : A data sequence $\mathbf{x_1}, \dots \mathbf{x_n}$ where $\mathbf{x_i} \in \mathbb{R}^d$
A measure of fit $\mathcal{C} : \mathbb{R}^d \rightarrow \mathbb{R}$.
A constant penalty term β
output: The change points of the data sequence recorded in $cp(n)$

Initialize $F(0) = -\beta,\ cp(0) = NULL,\ R_1 = \{0\}$
for $i \leftarrow 1$ **to** n **do**
  $j^* \leftarrow \arg\min_{j \in R_i} [F(j) + \mathcal{C}(\mathbf{x_{j+1:i}}) + \beta]$
  $cp(i) \leftarrow [cp(j^*), j^*]$
  $R_{i+1} \leftarrow \{j \in R_i \cup \{i\} \ : \ F(j) + \mathcal{C}(\mathbf{x_{j+1:i}}) \leq F(i)\}$
end

Hidden semi-Markov models

To complement the short description of the hidden semi-Markov models in Section 2.3 of the main paper, we provide a more formal definition of such models in the following. Formally, an HSMM is a tuple $\theta = (\mathcal{S}, \mathcal{O}, \Pi, P, B, D)$, where $\mathcal{S}$ is the state set, $\mathcal{O}$ is the observation space, $\Pi = \{\pi_j \mid j \in \mathcal{S}\}$ is the *initial state distribution*, $P = \{p_{i,j} \mid (i,j) \in \mathcal{S} \times \mathcal{S}\}$ is the *transition model*, $B = \{b_j(o) \mid j \in \mathcal{S}, o \in \mathcal{O}\}$ is the *emission model* and $D = \{d_j(u) \mid j \in \mathcal{S}, u \in \mathbb{N}\}$ is the *duration model*. The latter allows to explicitly model the distribution over the number of time steps the system stays in any given state, which is what distinguishes HSMMs from the simpler hidden Markov models (HMMs). In HMMs, these distributions are constrained to be geometric and are not explicitly modeled.

Inference in HSMMs can be performed in a similar way as in HMMs. First, one iteratively computes maximum-likelihood estimates for the parameters of the HSMM using the Baum-Welch algorithm. Second, one computes the most probable state sequence given the observations, i.e., the maximum-a-posteriori (MAP) using the Viterbi algorithm [1]. In our setting, after performing inference on a set of surgical trajectories, the MAP sequence for a specific surgical trial is the sequence of activities $\boldsymbol{A}$ that best describes the observed data.

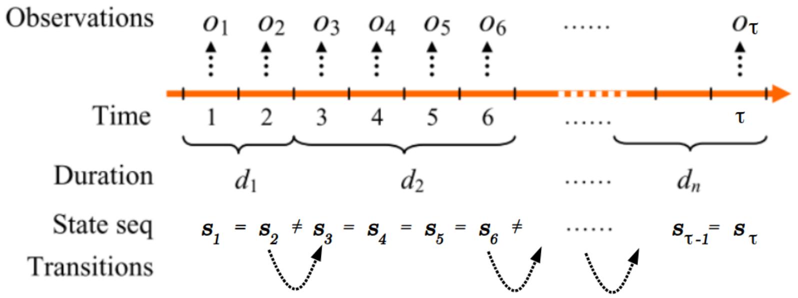

Fig. S2. Visualization of a HSMM and its data generating process: A dynamic system described by a HSMM remains in a state s for a duration d that is sampled from its duration model and then transitions to another state s' sampled from the transition model. The state sequence is not observed. Instead one only observes noisy observations o that depend on the state s of the system and are sampled according to the emission model. Adapted from [6].

Description of the observables

A variety of different categorical observables was extracted from the continuous and categorical sensor data. The super set $\mathcal{O}$ defined by all observables and their respective realization spaces (domains) as given in the Table S4 defined the observation space. The observables used for the experiments in the paper and referred to in Section 6 of the main paper are described in Table S4.

Observable	Description	Domain
coagulation_pedal	An indicator if the pedal that applies voltage to the hysteroscope such that it can be used to coagulate is active.	{On, Off}
cutting_pedal	An indicator if the pedal that applies voltage to the hysteroscope such that it can be used to cut is active.	{On, Off}
handle_movement	The description of the movement of the extraction of the hysteroscope.	{forward, still, backward}
pedals_yet_activated	An indicator if one the pedals has already been activated in the past part of the surgery.	{True, False}
turbidity	An indicator if the camera view is turbid (as a result of bleeding after cuts).	{True, False}
end_of_diagnosis	The indicator if the first change point of the update gate 14 is reached, which marks the end of the diagnosis.	{True, False}
seen_last_pedal_use	The indicator, if no pedal will be used in the remainder of the procedure.	{True, False}

Tab. S4. Overview of the observables derived from the sensor measurements and the self-supervised learning approach.

References

[1] Guédon, Y.: Hidden hybrid markov/semi-markov chains. Computational statistics & Data analysis **49**(3), 663–688 (2005)

[2] He, K., Zhang, X., Ren, S., Sun, J.: Deep residual learning for image recognition. In: Proceedings of the IEEE conference on computer vision and pattern recognition, pp. 770–778 (2016)

[3] Killick, R., Fearnhead, P., Eckley, I.A.: Optimal detection of changepoints with a linear computational cost. Journal of the American Statistical Association **107**(500), 1590–1598 (2012)

[4] Krizhevsky, A., Sutskever, I., Hinton, G.E.: Imagenet classification with deep convolutional neural networks. In: Advances in neural information processing systems, pp. 1097–1105 (2012)

[5] Twinanda, A.P., Yengera, G., Mutter, D., Marescaux, J., Padoy, N.: Rsdnet: Learning to predict remaining surgery duration from laparoscopic videos without manual annotations. IEEE transactions on medical imaging **38**(4), 1069–1078 (2018)

[6] Yu, S.Z.: Hidden semi-markov models. Artificial intelligence **174**(2), 215–243 (2010)
